# Accuracy improvement *via* novel ratiometry design in distance-based microfluidic paper based analytical device: instrument-free point of care testing†

**DOI:** 10.1039/d3ra01601c

**Published:** 2023-05-23

**Authors:** Sabah H. Al-Jaf, Khalid M. Omer

**Affiliations:** a Department of Chemistry, College of Science, University of Sulaimani 46002 Sulaimani City Kurdistan Region Iraq khalid.omer@univsul.edu.iq; b Department of Chemistry, College of Science, University of Garmian Darbandikhan Road 46021 Kalar City Sulaimaniyah Province Iraq; c Center of Biomedical Analysis, Department of Chemistry, College of Science, University of Sulaimani 46002 Sulaimani City Kurdistan Region Iraq

## Abstract

Developing accurate, precise, instrument-free, and point-of-need microfluidic paper-based devices is highly significant in clinical diagnosis and biomedical analysis. In the present work, a ratiometric distance-based microfluidic paper-based analytical device (R-DB-μPAD), along with a three-dimensional (3D) multifunctional connector (spacer), was designed to improve the accuracy and detection resolution analyses. Specifically, the novel R-DB-μPAD was used for the accurate and precise detection of ascorbic acid (AA) as a model analyte. In this design, two channels were fabricated as detection zones, with a 3D spacer located between the sampling and detection zones to improve the detection resolution by preventing the reagents mixing from overspreading between these zones. Two probes for AA were used: Fe^3+^ and 1,10-phenanthroline were deposited in the first channel, and oxidized 3,3′,5,5′-tetramethylbenzidine (oxTMB) was added to the second channel. Accuracy improvement of this ratiometry-based design was achieved by enhancing the linearity range and reducing the volume dependency of the output signal. Moreover, the 3D connector improved the detection resolution by eliminating the systematic errors. Under the optimal conditions, the ratio of the distances of the color bands in the two channels was used to construct an analytical calibration curve in the range from 0.05 to 1.2 mM, with a limit of detection of 16 μM. The proposed R-DB-μPAD combined with the connector was successfully used for the detection of AA in orange juice and vitamin C tablets with satisfactory accuracy and precision. This work opens the door for multiplex analysis of various analytes in different matrices.

## Abstract 2

Instrument-free and point-of-need microfluidic paper-based device for biomedical analysis.

## Graphical abstract

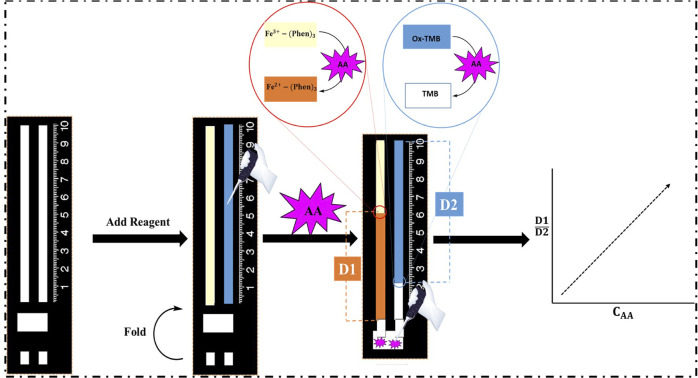

## 1. Introduction

Ascorbic acid (AA), also known as vitamin C, is essential for a variety of biological processes, such as reducing oxidative stress on the substrate of ascorbate peroxidase as a potent antioxidant.^1^ It plays a key role in many other processes – for example, it acts as a cofactor in enzymatic reactions and contributes to collagen synthesis and immune system enhancement.^2^ Maladjustment of or deficiency in AA content is associated with the symptoms of certain diseases, such as scurvy, mental illness, and cancer.^3^ Medical research has revealed that AA can be used to treat colds, mental disorders, infertility, cancer, and AIDS.^4^ AA can only be obtained from food intake to maintain the necessary levels for the human body.^3^ Thus, to monitor its content and guide medical diagnoses and dietary measures, the development of a reliable, selective, and simple method for AA determination in food safety and disease diagnosis is vital.

Currently, numerous analytical techniques developed for the determination of various biological molecules, such as electrochemistry,^5,6^ high-performance liquid chromatography,^7^ fluorescence,^8–12^ colorimetry,^13–17^ chemiluminescence,^18^ capillary electrophoresis,^19^ and the enzyme-linked immunosorbent assay (ELISA).^20,21^ However, most of these methods and techniques suffer from obstacles such as the need for costly and sophisticated instruments, skilled personnel, complicated pretreatments, or costly biological reagents. Therefore, finding an accurate and precise sensing platform to overcome the aforementioned limitations is highly desirable in bioanalysis.

Paper is an emerging solid support material that can be used in chemical and biological analyses due to its widespread availability, low costs, and the provision of a high-contrast background for colorimetric detection.^22^ Microfluidic paper-based analytical devices (μPADs), first reported in 2007 by Whitesides *et al.*, have evolved into analytical platforms that are well suited for resource-limited environments and on-site settings because they are easy to use, portable, and inexpensive.^23^ In addition, simple and rapid fabrication steps make these devices more attractive for worldwide users.^24^

Numerous detection techniques have been employed for quantitative analysis using μPADs, such as colorimetry,^25^ fluorescence,^26^ electrochemistry,^27^ chemiluminescence,^28^ electrochemiluminescence,^29^ and surface-enhanced Raman spectroscopy.^30^ These methods are still based on using some kinds of instruments such as cameras or scanners (when color or fluorescence is present) or potentiometers (for electrochemical designs), thus still necessitating an instrument for the analysis.^31–33^

Distance-based μPADs (DB-μPADs) constitute an advanced and simplified form of μPADs where no instrument is used for detection purposes.^34,35^ DB-μPADs are used to quantify analytes by measuring the length of a colored band (or the loss of color) that forms (or arises) along μPAD channels in the presence of the analyte.^35^ Additionally, employing imaging software to measure the length of colored bands yields fewer errors compared to using colorimetric detection alone.^36^

One of the problems in paper-based devices in general and specially in distance based detection (DB-μPADs) that needs to be addressed is the leaching of the reagents from the detection zone towards the sampling zone, which results in systematic errors. Thus, eliminating this source of errors is crucial, and relevant corrections must be made. Additionally, inconsistency of depositing sample volumes in the sampling zone is another issue that affects dramatically the signal output; hence another correction should be addressed.

Ratiometric assays, where the quantification of targets is based on the ratio of the intensity of two signals, have drawn significant attention in recent years. This technique can effectively reduce different kinds of instrumental or environmental errors and offers more accurate and precise analyses than assays based on a single signal, especially for targets with low concentrations or those in complex biological systems.^37,38^ Ratiometric measurement is based on the self-calibration of signal intensity *via* the recording of two or more analyte-induced signal fluctuations, where one signal can act as a reference factor for normalizing the others.^39^ In this light, implementing a ratiometric approach in DB-μPAD is highly important. To the best of our knowledge, prior research studies have not addressed combining ratiometry with DB-μPAD.

**Fig. 1:**
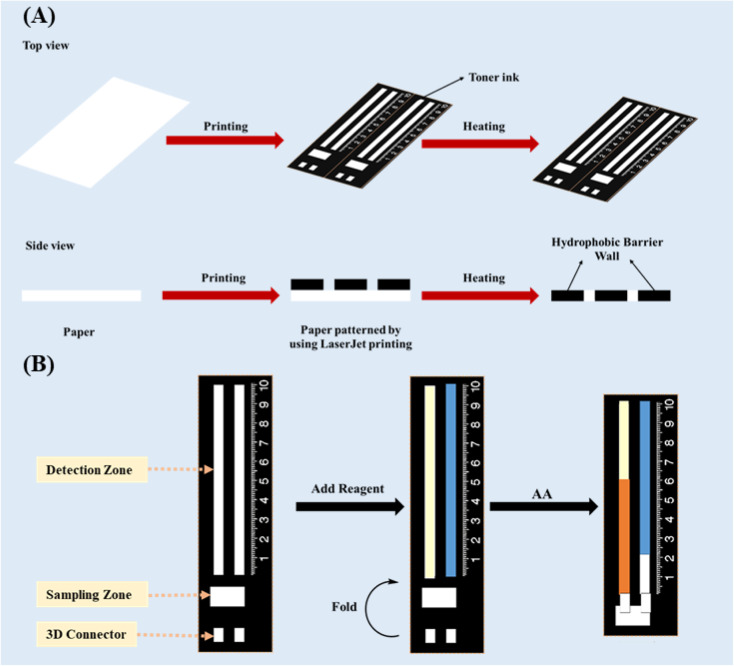
Schematic illustrations of (A) the procedure for the fabrication of the ratiometric DB-μPAD proposed herein, using a LaserJet printing approach; and (B) the sequence of steps to perform the measurements using this device.

In the present work, a ratiometric DB-μPAD (R-DB-μPAD) combined with a three-dimensional (3D) multifunctional spacer was constructed. The ratiometric method was designed to eliminate environmental fluctuations and irreproducibility in depositing the sample volumes. The 3D spacer was introduced between the sampling and detection zones to block the dispersion of reagents from the latter towards the former, resulting in improved detection resolution. The probes were based on the reducing properties of AA, reducing both the Fe^3+^–(phen)_3_ complex and the oxidised 3,3′,5,5′-tetramethylbenzidine (oxTMB) to Fe^2+^–(phen)_3_ (D1) and TMB (D2), respectively, where “phen” refers to 1,10-phenanthroline. Fig. 1 is a schematic illustration of the fabrication of the R-DB-μPAD proposed herein; the novel design was used for the quantification of AA in vitamin C tablets and orange juice. The proposed analytical device can be used to determine the concentration of AA in different samples, offering low costs, simplicity, and portability accompanied by high accuracy and precision.

## 2. Experimental

### 2.1 Materials

All materials and reagents utilized in this study were of analytical grade and used directly without further purification. 1,10-Phenanthroline (phen) and 3,3′,5,5′-tetramethylbenzidine (TMB) were purchased from Sigma-Aldrich (Sigma-Aldrich, Germany); sodium persulfate, ethanol, ascorbic acid, citric acid, glucose, fructose, sucrose, sodium chloride, potassium chloride, and calcium chloride from Biochem Chemopharma Chemical Industry Co., Ltd (Cosne Sur Loir, France); tyrosine, proline, alanine, histidine, arginine, and lysine from Carl Roth GmbH + Co. KG (Karlsruhe, Germany); and the orange juice and vitamin C tablets from a local market and a pharmacy, respectively, in Kalar, Sulaymaniyah (Iraq).

### 2.2 Design and fabrication of μPADs

The DB-μPAD was designed based on our previous work^40^ using Microsoft PowerPoint and printed on Whatman No. 1 filter paper. The device consisted of a rectangular reservoir (3 × 10 mm) for sample addition (sampling zone), as shown in Fig. 1A. Two straight parallel channels (3 × 60 mm) were fabricated as detection zones for the deposition of the Fe^3+^–(phen)_3_- and oxTMB-based probes. A hydrophobic space 3 × 3 mm in size was designed between the sampling and detection zones, and a 3D connector with the same dimensions was also developed. After printing the designed pattern onto the filter paper using a LaserJet printer (HP, M203), the paper was heated at 180 °C for 2 hours in an oven and then cooled to room temperature to ensure that the ink had completely penetrated the paper pores (Fig. 1A).

### 2.3 Deposition of probes and sample

After the fabrication process, the detection zones were functionalized with an appropriate amount of Fe^3+^–(phen)_3_ complex (probe 1, first channel) and oxTMB (probe 2, second channel), and the device was subsequently dried at room temperature for 5 minutes (Fig. 1B). During the analysis, the 3D spacer was aligned parallel to the detection and sampling zones by folding the connector; the sample or standard solution was then added to the sampling zone, which was then moved into the detection zone (Fig. 1B). Finally, the distance of the color band on both detection zones was measured, and the ratio of the distance of the first probe (D1) to that of the second probe (D2) was used to construct the analytical calibration curve and determine the concentration of AA.

#### 2.3.1 First probe

The color of the complex formed between Fe^3+^ and phen is pale yellow, which changes to orange-red when the Fe^3+^ is reduced to Fe^2+^. This principle was used herein as a probe for the detection of AA since AA can reduce Fe^3+^ to Fe^2+^,^41,42^ hence changing the color of the complex; the reaction between Fe^3+^–(phen)_3_ and AA is given in eqn (1) in Fig. 1S.† The first detection zone was saturated with the probe through the addition of 30 μL (3 × 10 μL increments) of Fe^3+^–(phen)_3_ by distributing it equally throughout the detection zone. After drying at room temperature, the valve between the detection and sampling zones was opened by simply folding the paper to align the 3D connector between these zones; 50 μL of the sample or standard solution of AA was then added into the sample reservoir. After coming into contact with the sample containing AA, the Fe^3+^–(phen)_3_ complex was reduced to Fe^2+^–(phen)_3_, resulting in the color change from pale yellow to orange-red.

#### 2.3.2 Second probe

TMB in its oxidized form (oxTMB) has a greenish-blue color, which upon the reduction of oxTMB to TMB, changes to colorless.^4,43^ Hence, we used oxTMB (obtained by oxidation of TMB by sodium persulfate) as the second probe in our design, which, in the presence of AA, is reduced to TMB, changing from greenish blue to colorless. This reduction reaction is shown in eqn (2) of Fig. 1S.† The second detection zone was first saturated with the probe through the addition of 30 μL (3 × 10 μL increments) of the oxTMB which equally distributed on the entire detection zone. After drying at room temperature, the valve between the detection and sampling zones was opened by simply folding the paper such that the 3D connector was well aligned between these zones; 50 μL of the sample or standard solution of AA was then added to the sample reservoir. Upon reaching the sample containing AA, the oxTMB was reduced to TMB, leading to the color change from greenish blue to colorless.

### 2.4 Sample pretreatment

The orange juice samples were filtered using 0.22 μm Millipore filters to remove the insoluble components and pulp and then diluted with ultrapure water. Regarding the vitamin C samples, 10 tablets were crushed to form a powder, 1 g of which was then dissolved in water and filtered through a 0.22 μm membrane.

## 3. Results and discussion

### 3.1 3D multifunctional connector and improvement of detection resolution

One of the main challenges in using DB-μPADs is controlling the flow of reagents from the detection towards the sampling zone during the deposition of reagents in the detection zone. The leaching of reagents into the sampling zone may lead to significant systematic errors since the consequent partial loss of analyte due to its reaction with the reagents cannot be accounted for. Thus, we designed the 3D connector (or spacer) in such a way that the detection and sampling zones remain disconnected until the deposition and drying of the reagents in the detection zone are complete. In this manner, we ensured no leaching of the reagents from the detection into the sampling zone. At a later stage, these zones can be connected by folding the paper such that the 3D space matches the hydrophobic space between the two zones (Fig. 1B), thus allowing the sample solution to flow easily from the sampling to the detection zone.

**Fig. 2:**
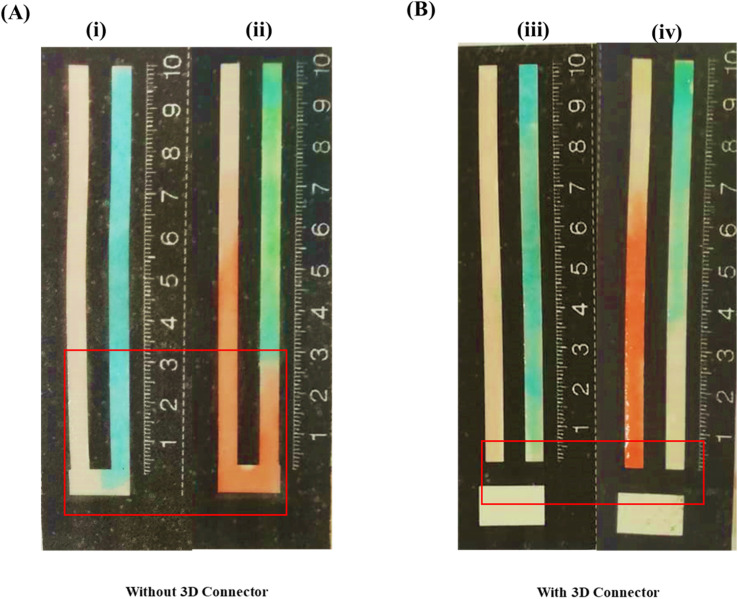
Distribution of the probes in the channels, using (A) the device without the 3D connector (i) before and (ii) after adding the sample solution and (B) the device with the 3D connector (iii) before and (iv) after adding the sample solution.

Fig. 2A and B depict the spreading of the probe solutions over the detection and sampling zones with and without the 3D connector. When no 3D connector is present (Fig. 2A), the deposited probe solutions spread (leach) into the sampling zone due to wicking, leading to a colored band developing in the sampling zone. As mentioned earlier, the leaching of the probes into the sampling zone causes significant errors since the consequent reaction of an amount of analyte with the probes will not be accounted for when measuring the distances of the color bands formed. In contrast, in the presence of a 3D connector (Fig. 2B), the probe solutions are distributed in the detection zone without any leaching into the sampling zone, thus leading to a well-defined colored band appearing in the former (Fig. 2A).

**Fig. 3:**
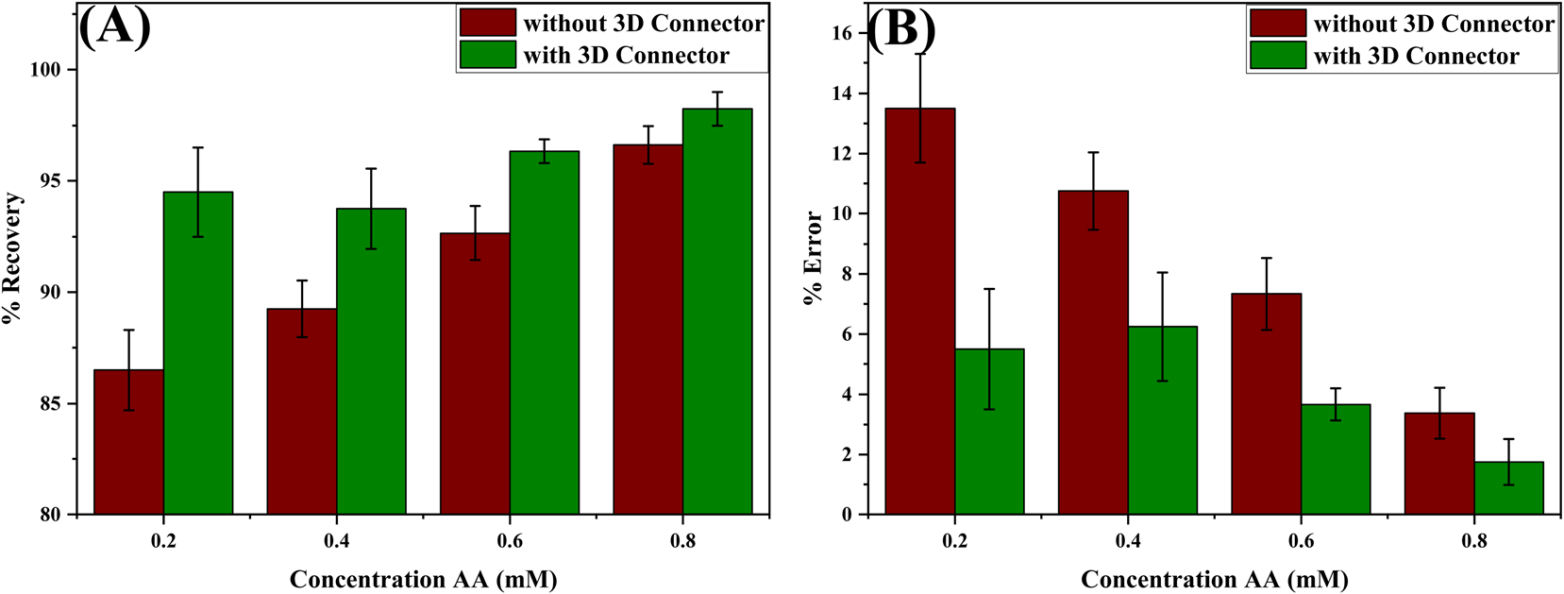
Variation of the (A) % recovery and (B) % error obtained for different standard AA solutions using the device with and without the 3D connector. The number of replications *n* = 3.

To evaluate the impact of the 3D connector on the accuracy of the obtained results, we conducted measurements on samples containing 0.2, 0.4, 0.6 and 0.8 mM AA using both designs, with and without the 3D connector. The recoveries obtained with the 3D connector are higher than those obtained without it, as shown in Table S1.† The percent error decreases from 8% for the 0.2 mM sample to only 1.62% for the 0.8 mM one, implying that the impact of the presence of the 3D connector is more significant in the case of lower concentrations of AA. Thus, we concluded that the 3D connector is highly important for detection resolution and minimizing errors in using DB-μPADs. The variations in the recoveries and percent errors for the different AA concentrations using the paper-based device with and without the 3D connector are shown in Fig. 3.

In addition to its role in connecting the detection and sampling zones, the 3D connector can be used as a masking or pretreatment zone in case the sample solution requires any pretreatment, seeing that it works as a bridge between the detection and sampling zones.

### 3.2 Ratiometric DB-μPADs

**Fig. 4:**
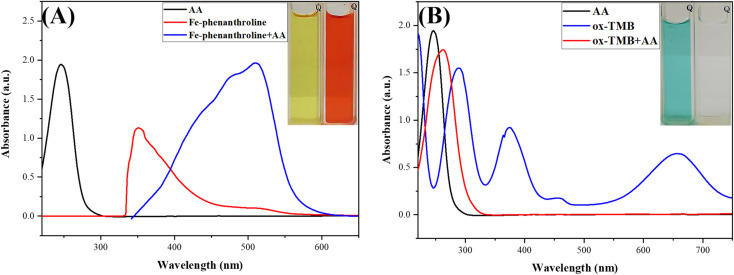
(A) UV-vis absorption spectra of ascorbic acid and the Fe^3+^–(phen)_3_ complex in the absence and presence of ascorbic acid; (inset) photograph of the Fe^3+^–(phen)_3_ complex in the absence and presence of ascorbic acid under visible light. (B) UV-vis absorption spectra of ascorbic acid and oxidised TMB (oxTMB) in the absence and presence of ascorbic acid; (inset) photograph of oxTMB in the absence and presence of ascorbic acid under visible light.

In this study's novel design, two chemical probes for the same target, AA, were deposited in the R-DB-μPAD channels. The first probe, a pale yellow-orange complex of Fe^3+^–(phen)_3_, changes to the orange-red Fe^2+^–(phen)_3_ upon the addition of AA due to the reduction of Fe^3+^ to Fe^2+^ ions. After optimizing the concentrations of Fe^3+^ and phen and based on the stoichiometry of the complex formed between them, 5 mL of 1.0 mM Fe^3+^ was mixed with 5 mL of 3.0 mM phen to form the Fe^3+^–(phen)_3_ complex. As shown in Fig. 4A, upon the addition of AA, a new absorption peak centered at 500 nm appears, and the peak centered at around 360 nm is weakened due to the reduction of Fe^3+^ to Fe^2+^ and the color change of the complex from pale yellow to orange-red.

The second probe in the detection zone, oxTMB, is blue in color. After the wicking of AA from the sampling to the detection zone, the blue color diminishes due to the reduction of oxTMB (blue) to TMB (colorless). Fig. 4B shows the spectra of oxTMB: the two peaks centered at around 380 and 650 nm in the absence of AA disappear upon the addition of AA due to the reduction of oxTMB to TMB and the color change from greenish blue to colorless.

After optimizing the concentrations of both probes, 30 μL (3 mM) of Fe^3+^–(phen)_3_ and 30 μL (2 mM) of oxTMB were added to the detection zones and left to dry at room temperature. Subsequently, after folding the paper to connect the detection and sampling zones, the samples or standards were added to the latter and moved towards the former due to capillary action. After reaching the detection zone, the AA reduced Fe^3+^ and oxTMB to Fe^2+^ and TMB, respectively, leading to the previously mentioned color changes. The whole process from the addition of the probes into the detection zones to addition of the samples and measuring the distance of the color band formed took about 10 min.

**Fig. 5:**
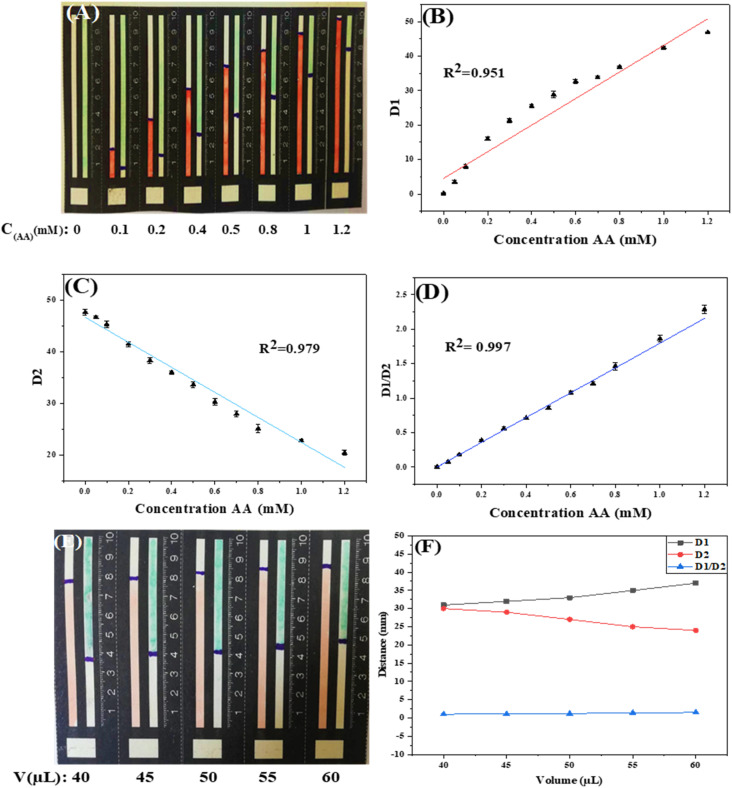
Distance-based measurements on the distance-based μPADs: (A) representative photographs of devices after exposure to standard solutions with increasing concentration of the analyte (from left to right); (B) and (C) analytical calibration curves of the measured distance of the first and second probes, respectively, determined separately as a function of the concentration of ascorbic acid; and (D) the analytical calibration curve of the ratio of the distances measured for the first (D1) and second (D2) probes as a function of the ascorbic acid concentration. (E) Representative photographs of the distance-based μPADs after exposure to different volumes of standard solutions AA (0.5 mM) with increasing the volumes from 40 to 60 μL (from left to right); (F) corresponding figure showing the separate distance-based signal for both probes and for the ratiometric signal.

Under the optimal conditions, the sensitivity and dynamic measurement range of the R-DB-μPAD design were evaluated. As shown in Fig. 5A, upon the addition of AA, the orange-red color of the Fe^3+^–(phen)_3_ probe gradually increases, while the greenish-blue color of the oxTMB probe gradually decreases.

Both probes can be used individually as single probes for the detection of AA as a linear relationship exists between D1 (the distance of the orange-red color) and AA (*R*^2^ = 0.951) and between D2 (the distance of the greenish-blue color of the oxTMB) and AA (*R*^2^ = 0.979), as shown in Fig. 5B and C.

To increase the linear range of our detection and minimize errors, we used the ratio of D1/D2 to construct the analytical calibration curve, as shown in Fig. 5D, displaying increased linearity of the curve (*R*^2^ = 0.997). The LOD of the proposed method was determined from the eqn (1).1
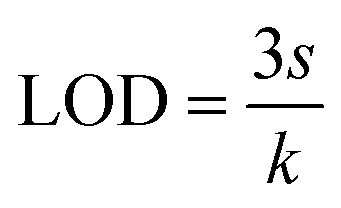
where *s* was the standard deviation of the blank sample and *k* was slope of the calibration curve in Fig. 5D. The calculated LOD was found to be 16 μM. As shown in Table 1, the figure of merit obtained for the proposed R-DB-μPAD combined with the 3D connector is comparable to those previously reported in the literature for the detection of AA using paper-based analytical devices.

**Table 1:** Comparison of AA detection herein with that in previously reported literature using paper-based analytical devices

Fabrication method	Sensing material	Detection method	Linear range (μM)	Limit of detection (μM)	Reference
Wax printing	KI/KIO_3_	Colorimetric	1–20	1.47	44
Wax printing	Carbon ink	Electrochemical	—	40	45
Laser printing	Graphite	Electrochemical	500–3000	70	46
—	AgNPs	Colorimetric	0–5677	2.27	47
Wax printing	AgNPs	Colorimetric	1000–4000	82.8	48
Wax printing	Prussian blue	Distance based	250–4000	440	49
Laser printing	Fe^3+^–(phen)_3_/TMB	Distance based	50–1200	16	Present study

**Fig. 6:**
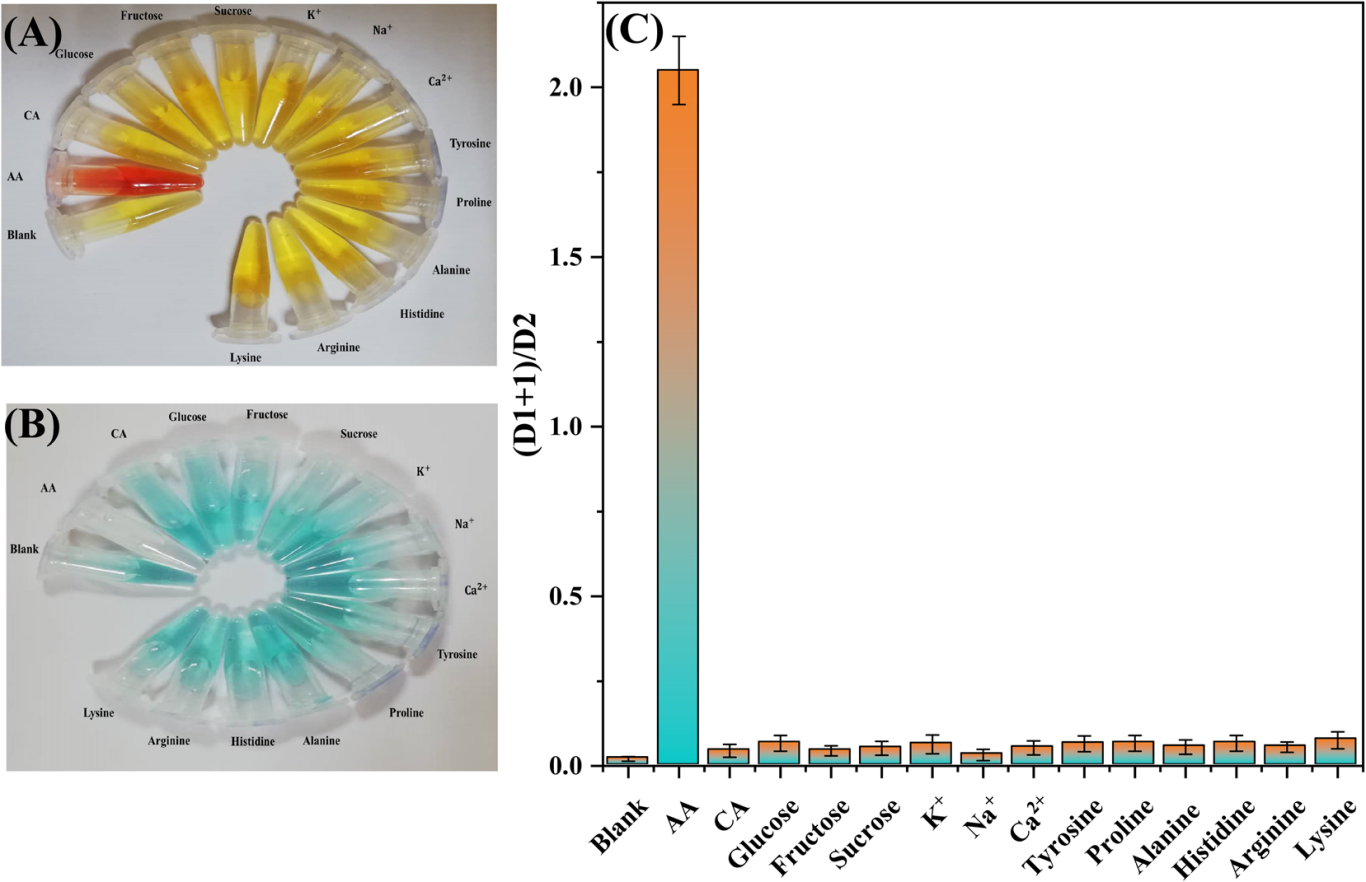
Selectivity of the selected methods towards AA: (A) and (B) selectivity of the first and second probes, respectively, and (C) selectivity on the distance-based μPAD using both probes. The concentrations of all substances are 0.5 mM.

The inherent dependence of the signal on the applied sample volumes is one of the key problems for colorimetric PADs in general, and notably for distance-based techniques. Despite the linearity improvement, the proposed ratiometric design can address this issue. Thus, using the ratiometric design any error is eliminated or minimized raised from using irreproducible volumes of the samples and/or standards. Fig. 6A and B show the impact of using different volumes of constant concentration of AA on the signals obtained for both probes, with and without the ratiometry design. As shown in Fig. 6B, the signals (elapsed distances) obtained without the ratiometry were varied if different volumes of the standard were used. While with the ratiometry mode, the signals obtained were remained constant even in case of using different volume of the standards. This concludes that adding slightly different volumes of samples or standards will not lead to significant variation in the obtained signal.

### 3.3 Selectivity study

To evaluate the selectivity of the proposed method, several potentially interfering molecules and metals commonly present in commercial orange juice and vitamin C tablets were employed. Possible interferences, such as glucose, fructose, sucrose, citric acid, tyrosine, proline, alanine, histidine, arginine, lysine, Na^+^, K^+^, and Ca^2+^, were prepared in a concentration of 0.5 mM and added to the sampling zone of the proposed design using the same procedure of applying the samples and standards. As shown in Fig. 6A and B, except for AA, none of the tested species had any significant response towards the Fe^3+^–(phen)_3_- and oxTMB-based probes. Therefore, we concluded that the proposed R-DB-μPAD combined with the 3D connector responds specifically towards AA without significant interference from other substances. The sensing platform is, thus, appropriate for the selective detection of AA in the orange juice and vitamin C tablet samples after dilution. The results of the selectivity test done by using the proposed design and using 0.5 mM of each of the tested interfering molecules and metals are shown in Fig. 6C.

### 3.4 Applications for real samples

To verify the reliability and potential application of the designed R-DB-μPAD combined with the 3D connector, the proposed analytical platform was used for the determination of AA in orange juice and vitamin C tablet samples. The pretreated samples were added into the sampling zone and the results for the unspiked samples were obtained. The accuracy of the proposed sensor was determined by adding different concentrations (0, 0.1, 0.2, and 0.3 mM) of AA standard solutions into each of orange juice and vitamin C tablet samples then added to the sampling zone to estimate their recoveries. As presented in Table 2, the recoveries of the known amounts of AA in the diluted real samples were between 82.03 and 107.87%, with the RSD ranging from 0.22 to 4.43%, indicating that the proposed sensor is applicable to the quantification of AA in orange juice and vitamin C tablets.

**Table 2:** Recovery tests in real orange juice and vitamin C tablet samples (*n* = 3) for the detection of the spiked AA using the proposed method

Sample	Unspiked (mM)	Spiked (mM)	Found (mM)	Recovery (%)	RSD (*n* = 3, %)
(1) Orange juice	0.59	0.1	0.67	82.03	1.06
		0.2	0.77	89.92	3.71
		0.3	0.89	98.41	3.24
(2) Orange juice	0.58	0.1	0.67	86.59	3.33
		0.2	0.78	99.55	3.05
		0.3	0.89	104.71	4.43
(3) Vit. C tablet	0.70	0.1	0.79	92.88	0.33
		0.2	0.89	94.79	3.97
		0.3	1.01	104.51	2.71
(4) Vit. C tablet	0.51	0.1	0.59	85.41	0.22
		0.2	0.71	101.21	3.22
		0.3	0.85	107.87	1.65

## 4. Conclusions

A novel design based on ratiometric distance-based microfluidic paper-based analytical device (R-DB-μPAD) combined with a 3D connector was developed and greatly improved the accuracy and detection resolution. The ratiometric distance based were successfully used for detection of AA based on its response to iron–phenanthroline complex and ox-TMB system. The R-DB-μPAD can eliminate environmental fluctuations and correct for sample volume irreproducible deposition in the sampling zone. The 3D connector significantly decreased the systematic errors from 15% to less than 1% by preventing reagents leaching from detection zones into the sampling zone. Both, the ratiometry and 3D connector design, contributed significantly to decrease the systematic and random errors within the analysis. Under the optimum conditions, the assay offers a good linear relationship between the Δ*A* signal and AA concentration in the range of 0.05–1.2 mM, with a detection limit of 0.016 mM. Notably, the proposed R-DB-μPAD combined with the 3D connector was successfully applied to the detection of AA in commercial orange juice and vitamin C tablets with high simplicity, feasibility, and reproducibility, illustrating its significant potential in the food and pharmaceutical industries. Based on this novel design, one can use this for a broad spectrum of analytes where more than one probe is available.

## Author contributions

Sabah H. Al-Jaf: investigation, formal analysis, writing original draft, validation. Khalid M. Omer: conceptualization, methodology, project administration, supervision, resources, writing-review and editing, visualization, validation.

## Conflicts of interest

Authors declare that there are no conflicts of interest with this submission.

## Supplementary Material

RA-013-D3RA01601C-s001
