# Supplementary material for: Accuracy improvement via novel ratiometry design in distance-based microfluidic paper based analytical device: instrument-free point of care testing
Source: RSC Adv. 2023 May 23;13(23):15704–13. doi: 10.1039/d3ra01601c (PMC10204734; doi:10.1039/d3ra01601c)
Supplement: RA-013-D3RA01601C-s001 [file RA-013-D3RA01601C-s001.pdf]

Electronic Supplementary Material (ESI) for RSC Advances.
This journal is © The Royal Society of Chemistry 2023

Electronic Supplementary Materials

Accuracy Improvement via Novel Ratiometry Design in Distance-Based Microfluidic Paper Based Analytical Device: Instrument-free POCT

Sabah H. Al-Jaf[a,b] and Khalid M. Omer[a]*

[a] Center of Biomedical Analysis, Department of Chemistry, College of Science, University of Sulaimani, 46002 Sulaimani City, Kurdistan region, Iraq

[b] Department of Chemistry, College of Science, University of Garmian, Darbandikhan Road, 46021, Kalar City-Sulaimaniyah Province, Kurdistan of Iraq

* Corresponding Email: khalid.omer@univsul.edu.iq

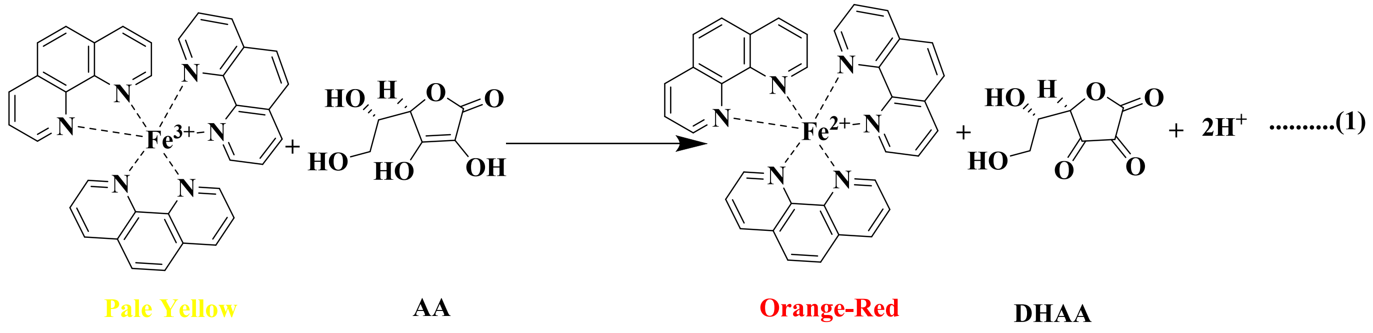

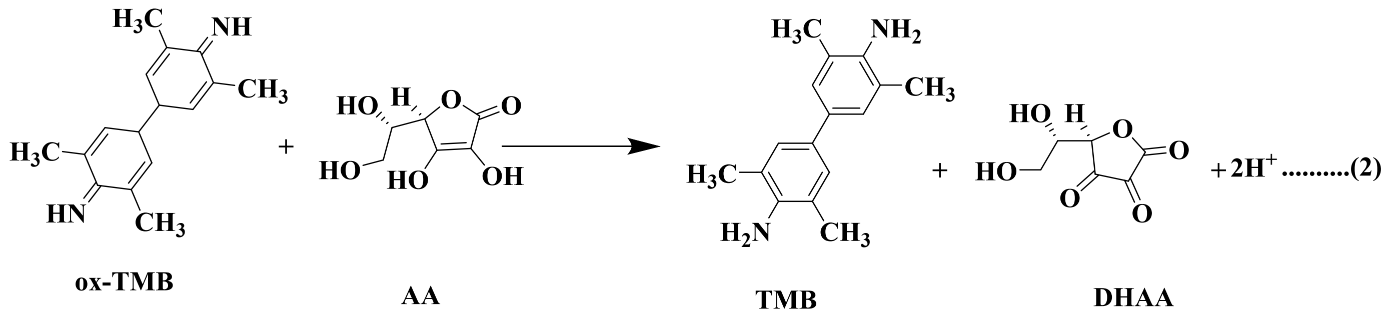

Figure S1. The reaction between Fe-1,10-Phenanthroline complex and AA (A), reduction of oxidized TMB to TMB in the presence of AA (B).

Table 1S

Comparison of the recoveries obtained for four different AA standards using the proposed distance-based design with and without a 3D connector.

Standard AA (mM)	Without 3D Connector			With 3D Connector			Difference in Error%
	Found (mM)	Recovery%	Error %	Found (mM)	Recovery%	Error%	
0.2	0.173	86.5 ± 1.8	13.5 ± 1.8	0.189	94.5 ± 2	5.5 ± 2	8
0.4	0.357	89.2 ± 1.2	10.7 ± 1.2	0.375	93.7 ± 1.8	6.2 ± 1.8	4.5
0.6	0.556	92.6 ± 1.2	7.3 ± 1.2	0.578	96.3±0.5	3.6 ± 0.5	3.67
0.8	0.773	96.6 ± 0.8	3.3 ± 0.8	0.786	98.2 ± 0.7	1.7 ± 0.7	1.62
